# Tanshinone IIA Alleviates Traumatic Brain Injury by Reducing Ischemia‒Reperfusion via the miR-124-5p/FoxO1 Axis

**DOI:** 10.1155/2024/7459054

**Published:** 2024-03-21

**Authors:** Wenbing Su, Meifen Lv, Dayu Wang, Yinghong He, Hui Han, Yu Zhang, Xiuying Zhang, Shaokun Lv, Liqing Yao

**Affiliations:** ^1^Rehabilitation Medicine of Qujing No. 1 Hospital, Qujing 655000, Yunnan, China; ^2^Department of Rehabilitation Medicine, The Second Affiliated Hospital of Kunming Medical University, Kunming 650000, Yunnan, China

## Abstract

**Background:**

Cerebral ischemia–reperfusion injury is a common complication of ischemic stroke that affects the prognosis of patients with ischemic stroke. The lipid-soluble diterpene Tanshinone IIA, which was isolated from *Salvia miltiorrhiza*, has been indicated to reduce cerebral ischemic injury. In this study, we investigated the molecular mechanism of Tanshinone IIA in alleviating reperfusion-induced brain injury.

**Methods:**

Middle cerebral artery occlusion animal models were established, and neurological scores, tetrazolium chloride staining, brain volume quantification, wet and dry brain water content measurement, Nissl staining, enzyme-linked immunosorbent assay, flow cytometry, western blotting, and reverse transcription–quantitative polymerase chain reaction were performed. The viability of cells was measured by 3-[4,5-dimethylthiazol-2-yl]-2,5-diphenyl tetrazolium bromide assays, while cell damage was measured by lactate dehydrogenase release in the *in vitro* oxygen glucose deprivation model. In addition, enzyme-linked immunosorbent assay, flow cytometry, western blotting, and reverse transcription–quantitative polymerase chain reaction were used to evaluate the therapeutic effect of Tanshinone IIA on ischemia/reperfusion (I/R) induced brain injury, as well as its effects on the inflammatory response and neuronal apoptosis, *in vivo* and *in vitro*. Furthermore, this study validated the targeting relationship between miR-124-5p and FoxO1 using a dual luciferase assay. Finally, we examined the role of Tanshinone IIA in brain injury from a molecular perspective by inhibiting miR-124-5p or increasing FoxO1 levels.

**Results:**

After treatment with Tanshinone IIA in middle cerebral artery occlusion–reperfusion (MCAO/R) rats, the volume of cerebral infarction was reduced, the water content of the brain was decreased, the nerve function of the rats was significantly improved, and the cell damage was significantly reduced. In addition, Tanshinone IIA effectively inhibited the I/R-induced inflammatory response and neuronal apoptosis, that is, it inhibited the expression of inflammatory cytokines IL-1*β*, IL-6, TNF-*α*, decreased the expression of apoptotic protein Bax and Cleaved-caspase-3, and promoted the expression of antiapoptotic protein Bcl-2. *In vitro* oxygen-glucose deprivation/reoxygenation (OGD/R) cell model, Tanshinone IIA also inhibited the expression of inflammatory factors in neuronal cells and inhibited the occurrence of neuronal apoptosis. In addition, Tanshinone IIA promoted the expression of miR-124-5p. Transfection of miR-124-5p mimic has the same therapeutic effect as Tanshinone IIA and positive therapeutic effect on OGD cells, while transfection of miR-124-5p inhibitor has the opposite effect. The targeting of miR-124-5p negatively regulates FoxO1 expression. Inhibition of miR-124-5p or overexpression of FoxO1 can weaken the inhibitory effect of Tanshinone IIA on brain injury induced by I/R, while inhibition of miR-124-5p and overexpression of FoxO1 can further weaken the effect of Tanshinone IIA.

**Conclusion:**

Tanshinone IIA alleviates ischemic–reperfusion brain injury by inhibiting neuroinflammation through the miR-124-5p/FoxO1 axis. This finding provides a theoretical basis for mechanistic research on cerebral ischemia–reperfusion injury.

## 1. Introduction

Every year, more than 40-million cases of disability are caused by the cerebral vascular rupture, ischemia, or stroke worldwide [1]. Currently, the leading cause of disability in adults is ischemic stroke, for which the primary treatment is the immediate restoration of blood supply [2]. However, this may exacerbate brain damage caused by ischemia‒reperfusion injury [3]. Worldwide, cerebral ischemia–reperfusion injury (CIRI) is an important cause of stroke death, damaging human health, and inducing a heavy economic burden on society [4]. CIRI is a condition that occurs after surgery for cerebral atherosclerosis and cerebral ischemia, is particularly common in patients who have undergone arterial or intravenous thrombolysis and is characterized by high morbidity, mortality, and recurrence [5]. Primary injury during ischemia and secondary injury during reperfusion are the pathological processes of CIRI, and the mechanism of injury is very complex [6–8]. However, the molecular mechanisms of the formation of cerebral ischemia‒reperfusion injury are still unclear.

*Salvia miltiorrhiza* is a well-known traditional Chinese herb that has been used clinically for more than 1,000 years. It is extensively used to treat cardiovascular diseases and has latent beneficial effects on chronic renal failure, hepatitis and Alzheimer's disease [9]. The lipid-soluble diterpene Tanshinone IIA can be isolated from *Salvia miltiorrhiza* and is the major functional component of *Salvia miltiorrhiza*. Various pharmacological functions have been found, such as preventing atherosclerosis, protecting the heart, inhibiting platelet accumulation, and antitumor effects [10–13]. Several studies have indicated that Tanshinone IIA alleviates cerebral ischemic injury [14, 15]. Several studies have demonstrated the potential protective effect of Tanshinone IIA on preventing cerebral ischemia‒reperfusion injury [16]. For example, Tanshinone IIA ameliorated cognitive defects by inhibiting endoplasmic reticulum stress-induced apoptosis in APP/PS1 transgenic mice [17]. Tanshinone IIA sodium sulfonate alleviated cardiac injury by modulating the system of oxidation resistance, inflammation and bast dysfunction in atherosclerosis [18]. However, the specific mechanism by which Tanshinone IIA alleviates cerebral ischemia‒reperfusion injury needs to be further investigated in depth.

Gene therapy has emerged as a potential therapeutic option for ischemic stroke. MicroRNAs (miRNAs) are noncoding small RNAs that target the 3ʹUTR of mRNAs to degrade mRNAs and thus participate in various pathophysiological processes [19]. As key regulators of many cellular biological processes, miRNAs are widely expressed in the central nervous system and are involved in the cell proliferation, apoptosis, and cell scorching [20]. MiR-124 is by far the most common miRNA found in neurons. miR-124 has been reported to be involved in regulating the polarity of activated microglia and the shift in macrophages to the anti-inflammatory M2 phenotype [21]. Early expression of miR-124 inhibits reactive astrocytes to reduce ischemic core formation [22].

miRNAs can participate in various pathophysiological processes after cerebral ischemia by regulating the mRNA of target genes [23, 24]. Forkhead transcription factor O1 (FoxO1), one of the FOX family members, is expressed in neurons, heart, and muscle and can bind to miRNAs to regulate their stability and translation. The previous study by Shen et al. [25] “Rbfox-1 promotes CaMKII*α* expression and cerebral hemorrhage-induced secondary brain injury by breaking micro-RNA-124” suggests a potential role for FoxO1 in mediating the development of ischemic stroke. miR-124-5p is the mature form of miR-124, indicating a potential link between miR-124-5p and FoxO1 in an ischemic stroke. Furthermore, recent studies have shown that miR-124-5p can delay the progression of cerebral aneurysms by regulating FoxO1 [26], which shows that the regulation of FoxO1 by miR-124-5p is critical in the disease progression. This study will focus on whether Tanshinone IIA alleviates ischemic–reperfusion brain injury through the miR-124-5p/FoxO1 axis.

## 2. Materials and Methods

### 2.1. Establishment of a Cerebral Ischemia‒Reperfusion Model

Healthy SD rats (250–300 g) were purchased from the Hunan Slack Jingda Laboratory Animal Co., Ltd. All animal experiments were carried out according to the Guide for the Care and Use of Experimental Animals and were authorized by the Ethics Committee of Hunan Provincial People's Hospital. In this study, a short-term middle cerebral artery occlusion-reperfusion (MCAO/R) model was established [27]. To put it simply, after the rats were anesthetized by an intraperitoneal injection of sodium pentobarbital, an incision was made in the middle of the neck, and the tissue was separated layer by layer to expose and dissociate the right common carotid artery, internal carotid artery, and external carotid artery. A small incision was made in the external carotid artery near the heart, through which sutures were inserted into the common carotid artery, and then retrieved through the bifurcation of the common carotid artery into the internal carotid artery. After that, the suture was slowly delivered to the origin of the middle cerebral artery and inserted about 8.0 ± 0.5cm from the bifurcation of the common carotid artery. Finally, sutures were secured and the skin of the neck was closed. In Sham operation group, only blood vessels were removed. After 24 hr of reperfusion, neurological function was scored. Brain tissue was used for the subsequent experiments.

### 2.2. Treatments

The male SD rats in each group were randomly divided into the following groups (*n* = 20/group): (1) model group; (2) Sham-operated group; (3) MCAO/R + Tanshinone IIA (4 mg/kg) group; (4) MCAO/R + Tanshinone IIA (8 mg/kg) group; and (5) MCAO/R + Tanshinone IIA (12 mg/kg) group. Tanshinone IIA (cat. no. HY-N0135) was purchased from MedChemExpress LLC (Monmouth Junction, NJ, USA). After 10 min of cerebral ischemia, the drug was administered by caudal vein injection, and after reperfusion, the drug was injected again.

### 2.3. Neurological Function Score Assessment

After 24 hr of ischemia‒reperfusion, the neurological deficits of rats were scored by the Longa scoring system [28] as follows: normal (0), incomplete extension of the right front paw (1), turning to the left (2), falling to the left (3), incapability to walk by themselves, and low level of self-awareness (4).

### 2.4. Measurement of Brain Water Content

Twenty-four hours after reperfusion, the wet weight of the brain was determined. Then, the brain was dried at 105°C for 24 hr, and the dry weight was recorded. Brain water content (%) = (wet weight−dry weight)/wet weight × 100%.

### 2.5. Nissl Staining

Paraffin-embedded brain tissue sections were routinely dewaxed and stained with Nissl solution (E607316, Sangon Biotech, Shanghai, China). Subsequently, the sections were rinsed and stained, dehydrated with ethanol, and sealed with neutral gum for observation and analysis.

### 2.6. Terminal Deoxynucleotidyl Transferase dUTP Nick End Labeling (TUNEL) Staining

PBS was used to wash the brain tissue. Ethanol was used to dehydrate the tissues to prepare clear, waxed, and embedded sections. Dewaxing and double-distilled water treatment were performed. The samples were sealed after the TUNEL reaction solution was added and were photographed by a fluorescence microscopy. The number of TUNEL-positive cells was observed.

### 2.7. Tetrazolium Chloride (TTC) Staining

Twenty-four hours after the operation, the animals were deeply anesthetized by an intraperitoneal injection of pentobarbital sodium (40 mg/kg), and brain sections were immersed in TTC solution for 10 min at 37°C. After being stained, the normal tissues were dyed red, while the blocked tissues were dyed white. In addition, the infarct volumes were observed, and the infarct rates were calculated by an Image-Pro Plus 6.0.

### 2.8. Establishment of an *In Vitro* Oxygen Glucose Deprivation/Reoxygenation (OGD/R) Cell Model

As previously described by Liu et al. [27], 18-day-old embryos from the pregnant SD rats were used to isolate primary cortical neurons. Primary cortical neurons were treated in a neural basal medium. Primary cortical neuronal cells were stimulated with OGD to establish a cerebral ischemia *in vitro* model. Before the OGD/R test, the cells were incubated with Tanshinone IIA (5, 10 or 15 *μ*M) for 30 min. After being resuspended in DMEM (Gibco, Sangon Biotech, Shanghai, China), the cells were transferred to an oxygen deficit well and cultured in a gas mixture of 94% N_2_ and 5% CO_2_. Then, the neuronal cells were incubated in a normoxic incubator.

### 2.9. 3-[4,5-Dimethylthiazol-2-Yl]-2,5-Diphenyl Tetrazolium Bromide (MTT) Measurement

Cell activity was detected by the MTT assays. Primary cultured cortical neurons were inoculated in 96-well plates at a density of 1 × 10^4^ neurons per well and treated with OGD/R. In addition, MTT solution was added, and the insoluble formazan crystals were collected and dissolved in the dimethyl sulfoxide (DMSO) at 37°C for 4 hr. After the crystals were fully dissolved, the OD570 value was measured by modulating the enzyme label. The experiment was repeated three times independently.

### 2.10. Lactate Dehydrogenase (LDH) Assay to Assess Cell Damage

The levels of LDH release in cultured cell supernatants were measured using an LDH assay kit (Beyotime). Primary cultured cortical neuronal cells were inoculated into 96-well plates for LDH activity analysis. LDH reaction solution (100 *µ*L) was added and incubated for 30 min, and the OD value was assessed at 490 nm by a spectrophotometer (Epoch, USA).

### 2.11. Enzyme-Linked Immunosorbent Assay (ELISA)

The levels of TNF-a, IL-6, and IL-1*β* were quantified using ELISA kits (TNF-*α*, ab245059, Abcam; IL-6, ab259381, Abcam; IL-1*β*, KE10003. Proteintech). The sample was added to the well, biotin-labeled antibody was added, and the cells were incubated at 37°C for 2 hr. Then, horseradish peroxide-labeled streptavidin was added and incubated. Finally, the chromogenic solution was added for 0.5 hr. The reaction was terminated, and the OD value was measured at 450 nm.

### 2.12. Cell Transfection

Control miRNA (5ʹ-UUCUCCGAACGUGUCACGUTT-3ʹ), the miR-124-5p mimic (5ʹ-CGUGUUCACAGCGGAACCUUGAU-3ʹ), and the miR-124-5p inhibitor (5ʹ-AUCAAGGUCCGCUGUGAACACG-3ʹ) purchased from Sangon (Shanghai, China) and transfected into the neuronal cells using lipofectamine 2000 (Invitrogen, Carlsbad, CA, USA). Forty-eight hours after transfection, neuronal cells are collected for the subsequent experiments.

### 2.13. Flow Cytometry

As previously described by Wang et al. [29], flow cytometry was used to measure apoptosis. In addition, the cells were incubated in an Annexin V plus fluorescein isothiocyanate/propidine iodide kit, and then Annexin V and PI fluorescence were determined at the referenced emission wavelengths by a Beckman cytometer (BD Biosciences) and FlowJo software (V11).

### 2.14. Reverse TranscriptionQuantitative Polymerase Chain Reaction (RT‒qPCR)

In this study, we extracted total RNA from tissues and cells using a Total RNA Extractor (Sangon Biotech). A cDNA synthesis kit (Vazyme, Nanjing, China) was used to reverse transcribe 2 *μ*g of RNA into cDNA, which was then diluted 10 times. One microliter of the prepared cDNA was used for qPCR. All primers (Table 1) used in this study were designed with Premier 5.0. The confidence of the qPCR results was assessed by the dissociation curve and cycle threshold (CT) values. The results were calculated by the 2^−*ΔΔ*Ct^ method and repeated at least three times.

### 2.15. Western Blotting

In this study, the proteins were extracted, and a BCA assay (Sangon Biotech, Shanghai) was used to determine the total protein content. A 10% SDS‒PAGE gel was used to separate the total proteins, which were then transferred to PVDF membranes by a constant current flow at 200 mA. Subsequently, the PVDF membranes were incubated with Bax, Bcl-2, and Cleaved-caspase-3 antibodies (Abcam, USA) for 12 hr at 4°C. The PVDF membranes were washed with TBS buffer and incubated with secondary antibodies (Abcam) at 25°C for 1 hr. After the membranes were washed three times, chemiluminescent reagents were added, and the grayscale values of the bands were analyzed using ImageJ software. Each experiment was repeated three times independently.

### 2.16. Dual Luciferase Reporter Gene Assay

The selected vector (Promega, Madison, WI, USA) was connected with the FoxO1 3ʹ-UTR sequence containing the binding site of miR-124-5p and the binding site sequence to be mutated. Then, the mutant (MUT) and original (WT) FoxO1 plasmids were constructed and transfected into the cells using Lipofectamine 2000 reagent with miR-124-5p mimics or miR-negative controls (NC). Then, a dual-luciferase reporter gene assay system was used to detect luciferase activity 48 hr after transfection.

### 2.17. Statistical Analysis

The data are presented as the mean ± SD. The difference between the two groups was compared using Student's *t*-test, and the comparison of differences between multiple groups was using one-way analysis of variance (ANOVA). *P* < 0.05 is considered statistically significant.

## 3. Results

### 3.1. Tanshinone IIA Pretreatment Attenuates I/R-Induced Brain Injury

**Figure 1:**
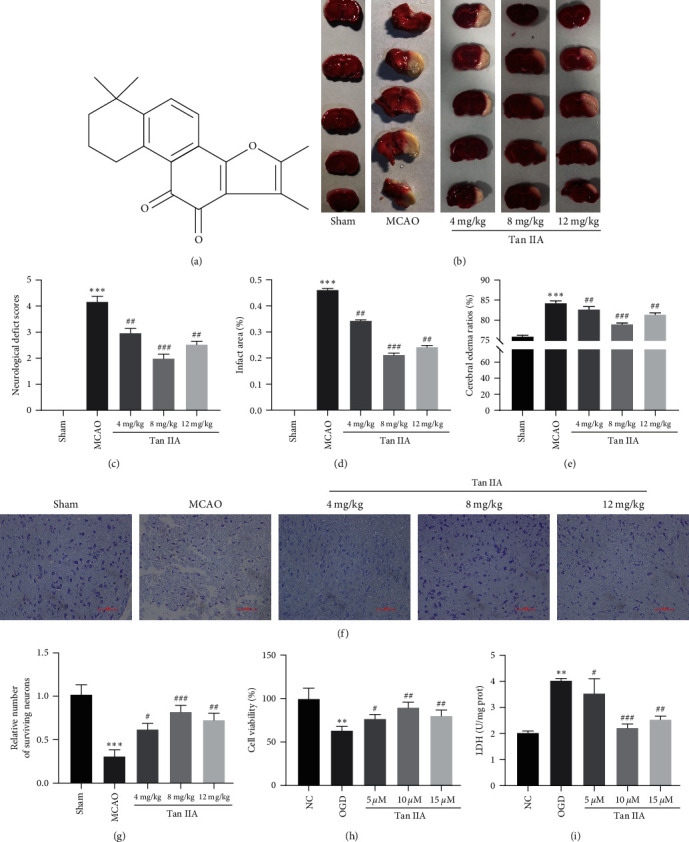
Tanshinone IIA pretreatment reduces I/R-induced brain injury. (a) Chemical structure of Tanshinone IIA; (b) TTC staining of the brain infarct area; (c) neurological scores; (d) changes in brain infarct volume; (e) wet and dry brain water content; (f) Nissl analysis of surviving neurons in the rat cortex, scale bar = 100 *μ*m; (g) quantification of surviving neurons in the rat cortex; (h) MTT analysis of cell viability; (i) LDH assay of cell damage.  ^*∗∗*^*P* < 0.01,  ^*∗∗∗*^*P* < 0.001 compared with the Sham group or NC group; ^#^*P* < 0.05, ^##^*P* < 0.01, ^###^*P* < 0.001 compared with the MCAO group or OGD group.

The structure of Tanshinone IIA is shown in Figure 1(a). To investigate the therapeutic affect of Tanshinone IIA on I/R-induced brain injury, the cerebral infarction volumes of rats were measured by TTC staining. The results showed that the brain infarct volume was increased in the MCAO group and decreased after treatment with Tanshinone IIA, and the best therapeutic effect was achieved in response to 8-mg/kg Tanshinone IIA (Figure 1(b)).The neurological deficits of rats were scored using a Longa scoring system, and the results showed that the MCAO group exhibited more severe neurological damage than the Sham-operated group, and neurological function was significantly improved after treatment with Tanshinone IIA (Figure 1(c)). At the same time, the results showed that the cerebral infarct volume decreased after Tanshinone IIA treatment, and the brain water content also decreased significantly (Figures 1(d) and 1(e)). We subsequently determined the protective effect of Tanshinone IIA on neurological function by Nissl staining. The results showed that the MCAO group exhibited abnormal neuronal size, increased cytoplasmic eosinophils, and visible vacuoles compared to the Sham-operated group (Figures 1(f) and 1(g)). Finally, the effects of Tanshinone IIA on cell proliferation and toxicity in OGD-treated neurons were evaluated *in vitro*; the MTT assay revealed that Tanshinone IIA altered cell activity (Figure 1(h)), and the LDH assay showed a significant decrease in cell damage after Tanshinone IIA treatment (Figure 1(i)). The best neuronal cell viability and the lowest cell damage were observed in response to 10 *µ*M Tanshinone IIA in conclusion, Tanshinone IIA pretreatment can reduce I/R-induced brain injury.

### 3.2. Tanshinone IIA Attenuates the I/R-Induced Inflammatory Response and Neuronal Cell Apoptosis

**Figure 2:**
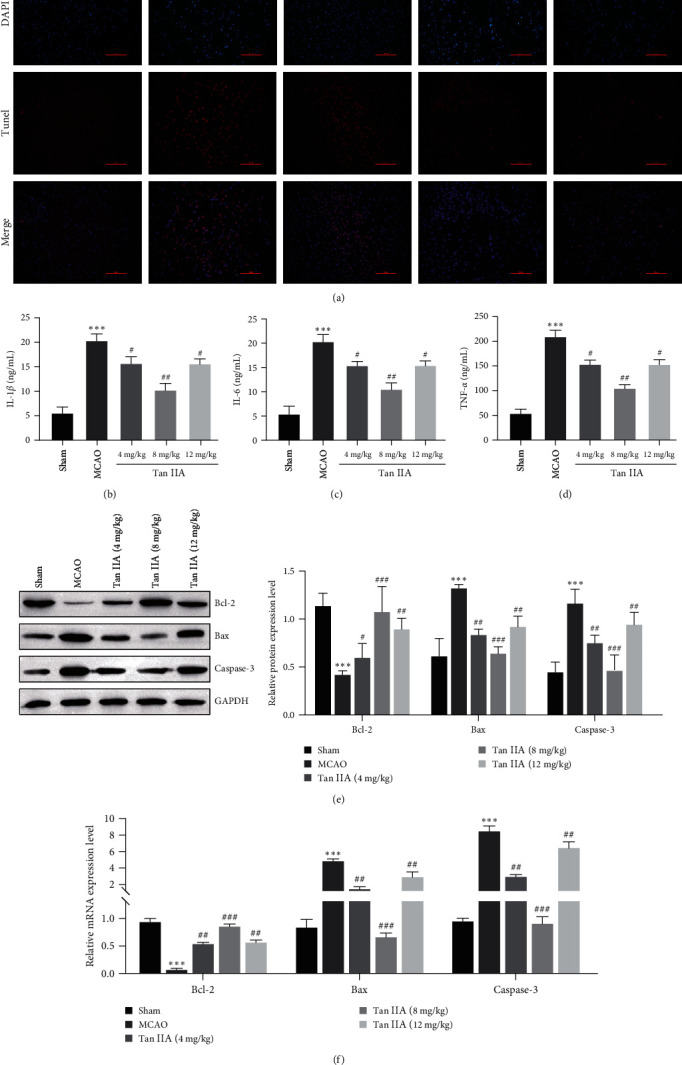
Tanshinone IIA reduces the I/R-induced inflammatory response and neuronal cell apoptosis. (a) TUNEL assay of apoptosis in rat cortical cells, scale bar = 100 *μ*m; (b–d) ELISA analysis of IL-1*β*, IL-6, and TNF-*α* levels; (e) apoptosis-related proteins were examined by western blot; (f) RT-qPCR was used to analyze the mRNA expression of apoptosis related proteins.  ^*∗∗∗*^*P* < 0.001 compared with the Sham group; ^#^*P* < 0.05, ^##^*P* < 0.01, ^###^*P* < 0.001, compared with the MCAO group.

To ascertain whether the mechanism by which Tanshinone IIA protects neurons affects inflammation and neuronal apoptosis, we examined an *in vivo* model by TUNEL, ELISA, western blotting, and RT–qPCR. Apoptosis was significantly increased in the MCAO group, and this effect was reversed by Tanshinone IIA (Figure 2(a)). The cytokines in rats were detected by ELISA. The levels of proinflammatory cytokines IL-1*β*, IL-6, and TNF-*α* in MCAO group were higher than those in the Sham group. Tanshinone IIA treatment significantly inhibited the production of proinflammatory cytokines (Figure 2(b)–2(d)). Compared with the Sham group, the expression of apoptosis-related proteins Bcl-2 was decreased and the expression of Bax and Cleaved-caspase-3 was increased in the MCAO group, which was significantly reversed after Tanshinone IIA treatment (Figure 2(e)). The results of RT–qPCR were consistent with those of the western blot (Figure 2(f)). These results indicated that Tanshinone IIA could reduce I/R-induced inflammation and neuronal apoptosis in an *in vivo* model.

### 3.3. Tanshinone IIA Attenuates the I/R-Induced Inflammatory Response and Neuronal Cell Apoptosis *In Vitro*

**Figure 3:**
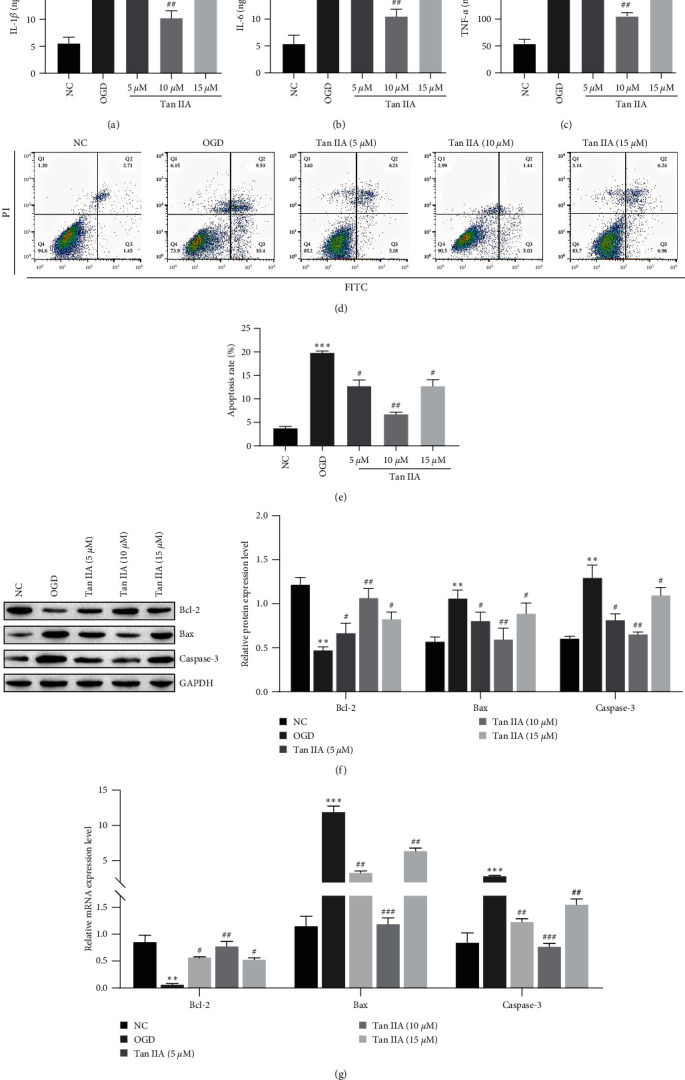
Tanshinone IIA attenuates the I/R-induced inflammatory response and neuronal cell apoptosis *in vitro*. (a–c) ELISA analysis of IL-1*β*, IL-6, and TNF-*α* levels; (d, e) flow cytometric analysis of apoptosis; (f) the expressions of apoptosis-related proteins were analyzed by western blot; (g) RT-qPCR was used to analyze the mRNA expression of apoptosis related proteins.  ^*∗∗*^*P* < 0.01,  ^*∗∗∗*^*P* < 0.001 compared with the NC group; ^#^*P* < 0.05, ^##^*P* < 0.01, ^###^*P* < 0.001 compared with the OGD group.

Previously, we determined that the protective effect of Tanshinone IIA on neurons was associated with inflammation and neuronal apoptosis *in vivo*. Next we examined neuronal cells. The data showed that the expressions of inflammatory factors IL-1*β*, IL-6, and TNF-*α* in the OGD group were significantly increased compared with the NC group, after Tanshinone IIA (5, 10, and 15 *μ*M) treatment, the expression of inflammatory factors IL-1*β*, IL-6, and TNF-*α* was significantly inhibited (Figure 3(a)–3(c)). These results demonstrate that the treatment with Tanshinone IIA has a robust protective effect against I/R-induced damage to the neuronal cells. Apoptosis was significantly promoted in the OGD group compared with the NC group and was significantly decreased after treatment with Tanshinone IIA (Figures 3(d) and 3(e)). Moreover, the level of apoptosis-related proteins was detected by the western blotting, and the results demonstrated that the OGD group had increased levels of Bax and Cleaved-caspase-3, and decreased levels of Bcl-2, treatment with Tanshinone IIA reversed the protein expression of Bax, Cleaved-caspase-3, and Bcl-2 (Figure 3(f)). The RT–qPCR results were similar to the western blot results (Figure 3(g)). Thus, treatment with Tanshinone IIA markedly attenuated alterations in apoptosis and the I/R-induced inflammatory response.

### 3.4. Tanshinone IIA Attenuates the I/R-Induced Inflammatory Response and Neuronal Apoptosis by Upregulating miR-124–5p

**Figure 4:**
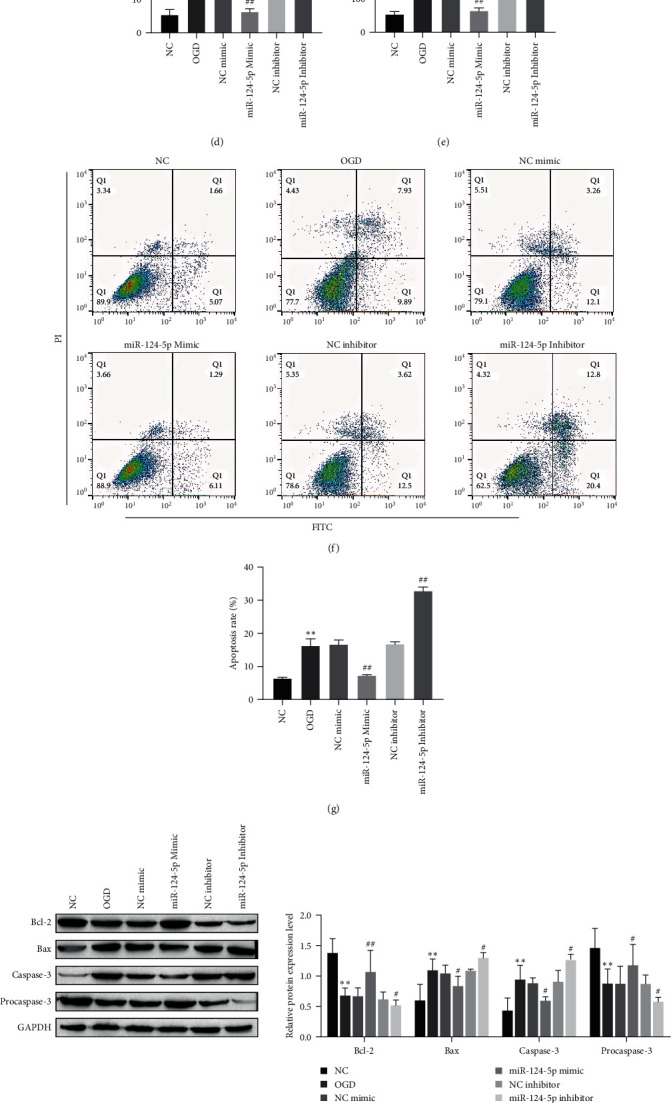
Tanshinone IIA attenuates the I/R-induced inflammatory response and neuronal apoptosis by upregulating miR-124-5p. (a) RT-qPCR was used to analyze the mRNA expression of miR-9, miR-124-5p, miR-132 and miR-148a-3p; (b) the transfection efficiency of miR-124-5p was analyzed by RT-qPCR; (c–e) ELISA analysis of cytokine expression; (f, g) flow cytometric analysis of apoptosis; (h) the expressions of apoptosis-related proteins were analyzed by western blot; (i) RT-qPCR was used to analyze the mRNA expression of apoptosis related proteins.  ^*∗*^*P* < 0.05,  ^*∗∗*^*P* < 0.01 compared with the NC group; ^#^*P* < 0.05, ^##^*P* < 0.01 compared with the OGD group.

Studies have reported that miRNA is involved in signal transduction and regulation of cerebral ischemia–reperfusion injury, which is a potential research object of neuronal injury and repair mechanism. In order to explore the potential molecular mechanism of Tanshinone IIA in alleviating cerebral injury of reperfusion, we detected several miRNAs (miR-9, miR-124-5p, miR-132, and miR-148a-3p) associated with neuron cells [30, 31]. RT–qPCR results showed that the expression of miR-124-5p, miR-132 and miR148a-3p in OGD group was down regulated compared with the NC group, except miR-9, after treatment with the Tanshinone IIA (10 *μ*M), the expression of miR-124-5p was most significantly increased (Figure 4(a)). The abundance of miR-124-5p on neurons may have a vital role in treating the ischemic brain injury, suggesting that miR-124-5p can be used as a marker of reperfusion-induced brain injury. To further validate the expression and the resulting effects of miR-124-5p on reperfusion injury, we evaluated the role of miR-124-5p in neuronal inflammatory response and apoptosis by transfecting miR-124-5p mimic, miR-124-5p inhibitor, and corresponding control (NC mimic, NC inhibitor) in the neuronal cells. First, the level of miR-124-5p increased or decreased significantly after transfection of the miR-124-5p mimic or miR-124-5p inhibitor, respectively (Figure 4(b)). Furthermore, ELISA detected that transfection of miR-124-5p mimic could inhibit the expression of proinflammatory factors IL-1*β*, IL-6, and TNF-*α*, but transfection of miR-124-5p inhibitor significantly promoted the expression of proinflammatory factors (Figure 4(c)–4(e)). Flow cytometry was used to detect cell apoptosis. The results showed that compared with the NC group, transfection with miR-124-5p mimic reduced the apoptosis of neuronal cells, while transfection with miR-124-5p inhibitor increased the apoptosis of neuronal cells (Figures 4(f) and 4(g)). The miR-124-5p mimic dramatically increased Bcl-2 expression but decreased Bax and Cleaved-caspase-3 expression, while the miR-124-5p inhibitor had the opposite effects (Figures 4(h) and 4(i)). Taken together, these results suggest that Tanshinone IIA attenuates the I/R-induced inflammatory response and neuronal apoptosis by upregulating miR-124-5p.

### 3.5. Validation of the Targeted Relationship between miR-124-5p and FoxO1

**Figure 5:**
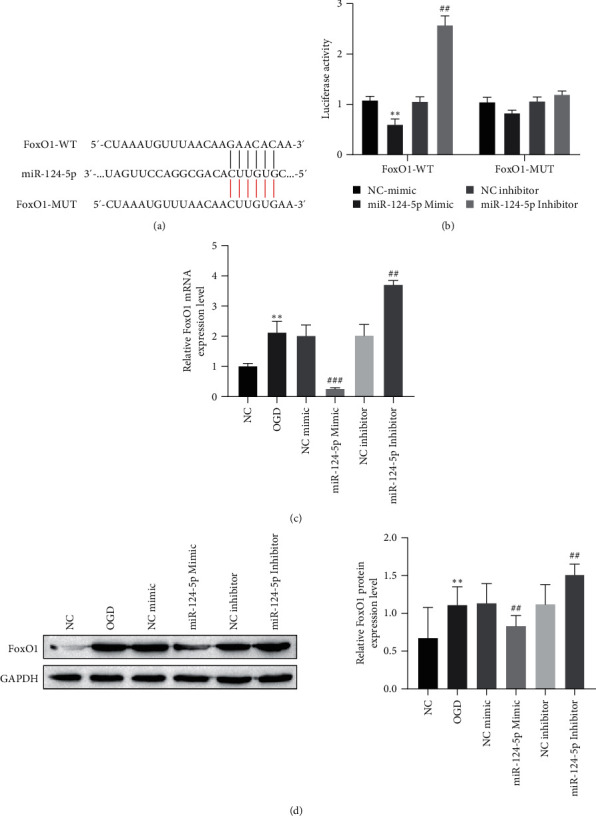
Validation of the relationship between miR-124-5p and FoxO1. (a) Target binding site; (b) dual luciferase assay; (c) the expression of FoxO1 was analyzed by RT-qPCR; (d) the expression of FoxO1 was analyzed by western blot.  ^*∗∗*^*P* < 0.01 compared with the NC-mimic group or NC group; ^##^*P* < 0.01, ^###^*P* < 0.001 compared with the OGD group.

According to the bioinformatics tool, miR-124-5p was predicted to have targeted binding sites with FoxO1 (Figure 5(a)). This relationship was examined using a dual luciferase reporter gene assay. MiR-124-5p mimic reduced the luciferase activity of FoxO1 wild type plasmid, while miR-124-5p inhibitor promoted the luciferase activity of FoxO1 wild type plasmid. However, miR-124-5p mimic and miR-124-5p inhibitor did not affect the luciferase activity of the mutant plasmid FoxO1 (Figure 5(b)). The miR-124-5p mimic dramatically reduced FoxO1 expression, and the miR-124-5p inhibitor significantly increased FoxO1 levels after OGD treatment (Figures 5(c) and 5(d)). Therefore, miR-124-5p clearly targets and downregulates an FoxO1 expression.

### 3.6. Inhibiting miR-124-5p or Increasing FoxO1 Eliminated the Inhibitory Effect of Tanshinone IIA on I/R-Induced Brain Injury

**Figure 6:**
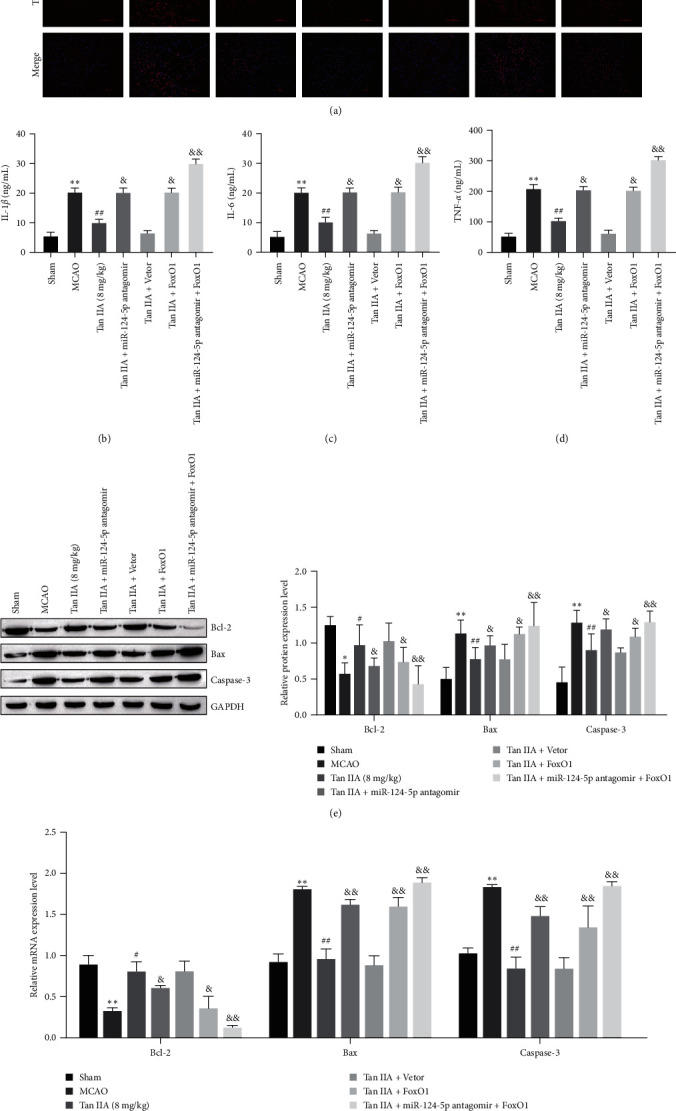
Inhibiting miR-124-5p or increasing FoxO1 abolished the inhibitory effect of Tanshinone IIA on I/R-induced brain injury. (a) TUNEL analysis of apoptosis in rat cortical cells, scale bar = 100 *μ*m; (b–d) ELISA analysis of IL-1*β*, IL-6, and TNF-*α* levels; (e) The expressions of apoptosis-related proteins were analyzed by western blot; (f) RT-qPCR was used to analyze the mRNA expression of apoptosis related proteins.  ^*∗*^*P* < 0.05,  ^*∗∗*^*P* < 0.01 compared with the Sham group; ^#^*P* < 0.05, ^##^*P* < 0.01 compared with the MCAO group; ^&^*P* < 0.05, ^&&^*P* < 0.01 compared with the Tan IIA group.

To further evaluate the role of miR-124-5p and FoxO1 in mediating Tanshinone IIA on neuronal apoptosis and inflammation. We observed the effect of miR-124-5p antiagomir or FoxO1 overexpression on the efficacy of Tanshinone IIA. TUNEL staining results showed that Tanshinone IIA could effectively reduce the occurrence of apoptosis, but miR-124-5p antiagomir or overexpression of an FoxO1 would attenuate the effect of Tanshinone IIA. Moreover, when miR-124-5p antiagomir acts with overexpression of FoxO1, the inhibitory effect on Tanshinone IIA is more significant (Figure 6(a)). The ELISA results showed that the miR-124-5p antagomir or FoxO1 overexpression markedly increased proinflammatory cytokines compared with the Tanshinone IIA group. MiR-124-5p antagomir and FoxO1 overexpression cotreatment further increased inflammatory factor levels in MCAO group (Figure 6(b)–6(d)). Western blot analysis showed that the miR-124-5p antagomir or FoxO1 overexpression treatment markedly increased the levels of the apoptotic proteins Bax, Cleaved-caspase-3, and reduced the level of Bcl-2 compared with the Tanshinone IIA group (Figure 6(e)). The results of RT–qPCR were consistent with those of western blot (Figure 6(f)). Taken together, Tanshinone IIA can regulate inflammation and apoptosis in the neuronal cells through the miR-124-5p/FoxO1 axis.

## 4. Discussion

Stroke is the second deadliest disease worldwide, and ischemic stroke is dominant at the time of onset. The sharp reduction in oxygen and nutrients in the ischemic core can mediate metabolic exhaustion, including adenosine triphosphate (ATP) depletion, membrane depolarization, and the overexpression of excitatory amino acids, which will eventually lead to irreversible neuronal damage [32]. Therefore, restoring blood flow to the ischemic location is the best way to reduce neuronal injury. However, thrombolytic reperfusion causes secondary neuronal injury and cellular inflammation through intricate signaling pathways that mediate neuronal death and survival [33]. Since reperfusion-induced neuronal injury and cellular inflammation are supposed to be beneficial, identifying protective remedies against reperfusion injury might be a strategy for the treatment of ischemic stroke. Our findings show that Tanshinone IIA alleviates the symptoms in the cerebral cortex of MCAO rats by reducing brain infarction, edema, neurological deficits, and neuronal apoptosis and inflammation.

Previous reports suggested that Tanshinone IIA, a monomeric chemical compound isolated from *Salvia miltiorrhiza*, has potential protective effects against cerebral ischemia‒reperfusion injury [34, 35]. Tanshinone IIA showed therapeutic effects on both the MCAO/R model and the OGD/R model, as evidenced by the reductions in neurological scores, a reduction in the area of cerebral infarction, and a reduction in brain water content in this study. The therapeutic effect was better when the concentration of Tanshinone IIA was 8 mg/kg and 10 *μ*M *in vivo* and *in vitro*, respectively.

The inflammatory response and apoptosis have important roles in reperfusion injury. The extensive activation of proinflammatory factors accelerates the progression of the inflammatory response [36, 37]. Excessive generation of oxygen free radicals creates excessive oxidative stress, which results cell and tissue damage, which is a major cause of acute cerebral ischemic injury [38]. Neuronal apoptosis is associated with the level of Bcl-2 family genes [39]. Caspase is central to apoptosis, and activated caspase-3 is the executor of apoptosis [40]. Caspase-3 guides cascade reactions by cleaving other caspase substrates, which ultimately results in apoptosis. Our results confirmed that Tanshinone IIA inhibited proinflammatory factors, including cytokines, and suppressed apoptosis, which were consistent with the previous studies [39].

As coding factors, miRNAs are widely activated in the central nervous system. These factors are vital regulators of many cellular biological processes, such as cell proliferation, apoptosis, and cell scorching. Microglia-derived exosome miR-124 suppresses neuronal inflammation after traumatic brain injury [41]. In addition, decreased miR-124 levels indicate neuroinflammation in various diseases, such as experimental autoimmune myelencephalitis [42] and cerebral hemorrhage [43]. A previous study showed that miR-124 injections promoted neuroprotection and functional healing during stroke episodes [22, 41]. In our study, we demonstrated that miR-124-5p was significantly downregulated in the OGD model, and the expression of miR-124-5p was significantly increased after the addition of Tanshinone IIA. Second, when we transfected the miR-124-5p mimic, the levels of proinflammatory factors and apoptosis were inhibited, while miR-124-5p inhibitor treatment enhanced the inflammatory response and increased apoptosis in the neural cells. This finding strongly suggests that Tanshinone IIA can attenuate the inflammatory response and neuronal apoptosis in reperfusion injury by upregulating the level of miR-124-5p.

To further clarify the molecular mechanism of Tanshinone IIA in the treatment of ischemic stroke, we predicted the targeted downstream factor of miR-124-5p by bioinformatics analysis. FoxO1 is expressed in neurons and is regulated by miRNAs [44]. It is exciting to note that miR-124-5p was also shown to target and downregulate an FoxO1 expression. FoxO1 is the first transcription factor 10 discovered in the FOXO family [45], which can participate in the regulation of various physiological and pathological processes of the cells by regulating related genes such as oxidative stress kinase, apoptosis, and autophagy [46, 47]. Meanwhile, it has been reported that FoxO1 can promote the development of cerebral ischemia/reperfusion (CI/R) injury [48]. In addition, studies have shown that artemisinin treatment can alleviate CIRI by mediating SIRT1/FoxO1 signaling pathway [49]. The expression of FoxO1 is upregulated in the brain tissue of CI/R-induced rat models [50]. Our study also showed that FoxO1 was highly expressed in the constructed CI/RI cell model. In addition, inhibition of miR-124-5p or overexpression of FoxO1 successfully weakened the protective effect of Tanshinone IIA on brain injury. These findings suggest that Tanshinone IIA ameliorates apoptosis and the inflammatory response in brain injury through the miR-124-5p/FoxO1 axis.

In conclusion, it was shown that the downregulation of miR-124-5p in MCAO and OGD models promotes FoxO1 expression, which leads to the neuronal cell apoptosis and inflammation. Tanshinone IIA alleviates ischemic–reperfusion injury via the miR-124-5p/FoxO1 axis, providing a new therapeutic target for ischemic stroke. Furthermore, our study has the limitation of not detecting changes in miR-124-5p and FoxO1 expression before and after Tanshinone IIA treatment in the clinical samples. In the next study, we will examine clinical samples to further verify the molecular indicators of this mechanism.

## Figures and Tables

**Table 1:** Fluorescent quantitative primer sequences.

Gene sequence	Primer name	Sequence (5′⟶3′)
Bax	Forward	AATATGGAGCTGCAGAGGATGA
	Reverse	CCCCCATTCATCCCAGGAAAA

Bcl-2	Forward	TCGCCCTGTGGATGACTGA
	Reverse	CAGAGACAGCCAGGAGAAATCA

Cleaved-caspase-3	Forward	ATGGAGAACAACAAAACCTCAGT
	Reverse	TTGCTCCCATGTATGGTCTTTAC

FoxO1	Forward	CACACAGTGTCAAGACTACA
	Reverse	CAAGACTTGAGACCATCACA

miR-124-5p	Forward	CGUGUUCACAGCGGAACCUUGAU
	Reverse	AUCAAGGUCCGCUGUGAACACG

U6	Forward	CTCGCTTCGGCAGCACATA
	Reverse	AACGCTTCACGAATTTGCGT

GAPDH	Forward	CCAGCTTAGGTTCATCAGG
	Reverse	GTGAAGACACCAGTAGACTC

## Data Availability

The data used to support the findings of this study are included within the article.
